# The Practitioner’s Guide to Global Health: an interactive, online, open-access curriculum preparing medical learners for global health experiences

**DOI:** 10.1080/10872981.2018.1503914

**Published:** 2018-08-07

**Authors:** Gabrielle A. Jacquet, Rachel A. Umoren, Alison S. Hayward, Justin G. Myers, Payal Modi, Stephen J. Dunlop, Suzanne Sarfaty, Mark Hauswald, Janis P. Tupesis

**Affiliations:** a Department of Emergency Medicine, Boston University School of Medicine, Boston, MA, USA; b Department of Pediatrics, University of Washington School of Medicine, Seattle, WA, USA; c Department of Emergency Medicine, Brown University School of Medicine, Providence, RI, USA; d Department of Emergency Medicine, University of North Carolina Chapel Hill, Chapel Hill, NC, USA; e Department of Emergency Medicine, University of Massachusetts Medical School, Worcester, MA, USA; f Department of Emergency Medicine, Hennepin County Medical Center, University of Minnesota, Minneapolis, MN, USA; g Department of Internal Medicine, Boston University School of Medicine, Boston, MA, USA; h Department of Emergency Medicine, University of New Mexico School of Medicine, Albuquerque, NM, USA; i Department of Emergency Medicine, University of Wisconsin School of Medicine, Madison and Public Health, University of Wisconsin - Madison, Global Health Institute, Madison, WI, USA

**Keywords:** Global health, international, MOOC, online, curriculum

## Abstract

**Background**: Short-term experiences in global health (STEGH) are increasingly common in medical education, as they can provide learners with opportunities for service, learning, and sharing perspectives. Academic institutions need high-quality preparatory curricula and mentorship to prepare learners for potential challenges in ethics, cultural sensitivity, and personal safety; however, availability and quality of these are variable.

**Objective**: The objective of this study is to create and evaluate an open-access, interactive massive open online course (MOOC) that prepares learners to safely and effectively participate in STEGH, permits flexible and asynchronous learning, is free of charge, and provides a certificate upon successful completion.

**Methods**: Global health experts from 8 countries, 42 institutions, and 7 specialties collaborated to create *The Practitioner’s Guide to Global Health* (PGGH): the first course of this kind on the edX platform. Demographic data, pre- and posttests, and course evaluations were collected and analyzed.

**Results**: Within its first year, *PGGH* enrolled 5935 learners from 163 countries. In a limited sample of 109 learners, mean posttest scores were significantly improved (*p* < 0.01). In the course’s second year, 213 sampled learners had significant improvement (*p* < 0.001).

**Conclusion**: We created and evaluated the first interactive, asynchronous, free-of-charge global health preparation MOOC. The course has had significant interest from US-based and international learners, and posttest scores have shown significant improvement.

## Background

Short-term experiences in global health (STEGH) [1,2] – including clinical rotations, research, language immersion, and volunteer work – are becoming more common at all levels of USA (US) medical education. Medical student participation in STEGH increased from 8% in 1986 to 31% in 2015 [3,4]. An estimated 74–80% of US emergency medicine residency programs reported at least one resident participating in a GH learning experience during the surveyed year [5,6]. Similar interest has been demonstrated in many other specialties [7–18].

The skills learned while participating in a STEGH may correlate with Accreditation Council for Graduate Medical Education competencies [19] and are associated with career choices focused on caring for underserved populations [20,21]. Despite these benefits, ethical concerns remain regarding individuals’ motives and unintended impacts on host institutions and populations [22], particularly with short-term experiences as they are often unsustainable and lack adequate follow-up and supervision [23–31].

Participation in STEGH also involves health and safety risks to trainees. Learners may find themselves in health-care systems and cultures with which they are unfamiliar and may be challenged to navigate high-risk situations involving ethics, personal safety, and cultural sensitivity [32–34]. Additionally, when learners return home, they may experience reverse culture shock and associated psychological stress. Robust guidance and adequate preparation for safe and effective STEGH are necessary to mitigate these risks, optimize the learning experience, and increase the chance of making useful contributions to the host population.

Global health curricula vary greatly in quality and cost – from no preparation at all to 3-week intensive in-person GH boot camps that often include case discussions, simulation, and focused workshops [35–37]. Online guides and simulation cases [38,39] provide relevant information but may not be organized in the most useful way and may require resources that make them less accessible than a massive open online course (MOOC); MOOCs are accessible by large numbers of learners around the world, run continuously, and provide an additional benefit of a learning community which persists after the course ends [40]. The purpose of this innovation was to create a free, timeline-based, interactive MOOC with the following objectives: (1) to prepare medical trainees to safely and effectively participate in STEGH, (2) to permit flexible, asynchronous learning in a MOOC with peers from around the world, and (3) to provide an electronic assessment so that completion of each part of the course may be tracked by program leadership. This study was conducted to evaluate whether an innovative, interactive, open-access online course could effectively prepare its enrolled learners for GH learning experiences.

## Methods

In 2014, GH faculty and learners were recruited on GH listservs and networks to participate in authoring a new MOOC for GH learners called *The Practitioner’s Guide to Global Health* (PGGH). Volunteers from 8 countries (Canada, India, Kenya, Lebanon, Moldova, South Africa, United Kingdom, and USA), 42 institutions, and 7 specialties were recruited over educational listservs and assigned to teams. The edX.org is an online learning destination and MOOC provider that offers high-quality courses from well-renowned universities and institutions to learners worldwide. The edX platform was chosen because it offers all of our desired features, is available in a phone ‘app,’ and can be updated easily. Course content was peer-reviewed by course advisors – physicians chosen for their extensive GH experience and reputation, and who had not already contributed to the course – and subsequently uploaded to the edX platform.

*PGGH* is the first timeline-based, interactive, evaluative course that helps prepare learners to participate in safe and ethical GH experiences. It is important to note that each part should be completed in conjunction with in-person faculty mentorship tracked by the learner’s home institution.

*PGGH* was first released in 2015 as a three-part open-access MOOC on the edX platform (www.edx.org) [41]. The production cost of this course is estimated at 75,000 USD – which falls within the quoted range of 39,000–325,300 USD – and the annual maintenance cost is considerably less [42].

The curriculum begins with an introduction to the edX platform, faculty introductions, and a pretest. The content is delivered through a multimodal approach, using text and key points, case studies, documentary-style video narratives, photographic images, practice questions, reflective exercises, and active participation in discussion forums to encourage active participation. The course contains interactive case scenarios, faculty-moderated discussion boards, and video vignettes by faculty and learners with GH experiences that illustrate common issues one may encounter in each phase of planning, participating in, and returning from GH experiences. These important topics – including exceeding one’s level of training, research ethics, use of photography, and dealing with requests for financial support – have been frequently reported by learners participating in GH experiences [33,43].

The *PGGH* curriculum is built on an extension of previously published timeline-based phases of a STEGH [44]: contemplation, preparation and participation, and reflection; as such, it is organized by topics and issues that may occur at different periods of time:

Part 1: ‘The Big Picture’ is to be completed 6–12 months before a STEGH and is relevant when trainees are contemplating whether/when/where to do a rotation. Part 1 asks the trainee to consider several important ‘Big Picture’ questions: Why do you want to have a STEGH? What kind of experience is appropriate for your current level of training? When and where should you do it? How will you fund it? Table 1 shows the syllabus for Part 1.

**Table 1.** Course objectives and syllabus for PGGH Part 1: The Big Picture.

**Objectives**	
1. Describe and prioritize your purpose and motivation for undertaking a GH experience	
2. Differentiate between different types of GH experiences and determine which ones provide the best fit for you	
3. Analyze factors including timing, duration, and location to plan an appropriate GH learning experience	
4. Develop a plan to address logistical issues including personal, health, and security concerns that affect successful completion of a GH learning experience	
5. Identify and describe options for funding and budgeting for a GH learning experience	
**Section**	**Content**
Course introduction	Course overview
	edX walkthrough
Introduction	Part 1 Overview
Why?	Purpose and motivation
	Mentors
	Ethics and social justice
	Risks and benefits

What?	Structure and design of global health experiences
	Specific scenarios

When?	Timing of experience
	Duration of experience

Where?	Site safety
	Housing conditions
	Culture and politics
	Language
	Sustainability

How?	Overview of logistics
	Security, travel, and communication
	Personal
	Academic and professional

Funding	Overview of funding
	Sources of funding
	Budgeting


GH: Global health.

Part 2: ‘Preparation and On The Ground’ is to be completed 1–3 months in advance of the STEGH and provides a nuts and bolts ‘how’ toolkit for predeparture preparation and on-the-ground experiences. Part 2 addresses the logistics of planning, health, cultural awareness and sensitivity, and dealing with unexpected situations while abroad. Table 2 shows the syllabus for Part 2.

**Table 2.** Course objectives and syllabus for PGGH Part 2: Preparation and On The Ground.

**Objectives**	
1. Effectively prepare for and arrange airport transportation and travel documents for a smooth arrival	
2. Improve cultural awareness and security preparedness in the areas of housing insurance, money, and clothing and evacuation	
3. Identify proper vaccinations and medications to limit health hazards	
4. Prepare an appropriately inclusive yet ‘light’ packing list that ensures preparation for emergencies and environmental exposures	
5. Describe practical strategies for an enriched educational experience that benefits you and your host community	
6. Navigate and manage personal and family responsibilities	
7. Identify and avoid common medical issues that you may encounter on the ground	
8. Recognize personal and property safety risks, including risks related to transportation and to drug and alcohol consumption	
9. Identify professional, ethical, and cultural issues you may encounter	
10. Use various modes of communication, including social media, responsibly	
**Section**	**Content**
Preparation: logistics	Overview of logistical preparation (personal safety, insurance, money, emergency action plan, transportation, communication)
	Safety, transportation, and communication logistics
	Personal logistics (vaccinations, malaria and postexposure prophylaxis, mental health, family health, traveling as a couple)
	Academic and professional logistics

Preparation: to serve and to learn	Cultural sensitivity and differences
	Professionalism and ethics
	Educational experience

On the ground: logistics	Overview of logistics on the ground
	Safety, transportation, and communication logistics
	Personal logistics
	Academic and professional logistics

On the ground: serving and learning	Cultural competency and differences
	Professionalism and ethics
	Educational experience

On the ground: unexpected circumstances	In case of emergency

Part 3: ‘Reflection’ is to be completed toward the end of the learner’s rotation or within 2 weeks of return from their STEGH to their home country. Part 3 helps the learner reflect on the challenges of returning home, dealing with unexpected physical and mental health issues, and planning for future work and sustainability. Table 3 shows the syllabus for Part 3.

**Table 3.** Course objectives and syllabus for PGGH Part 3: Reflection.

**Objectives**	
1. Identify and explain components of ‘reverse culture shock’ upon returning from a global health experience	
2. Identify strategies for effectively ‘reintegrating’ into your home and work life upon returning from a global health experience	
3. Effectively deal with potential health issues upon returning from a global health experience	
4. Effectively advocate for other individuals at your institution to identify clinical opportunities, educational opportunities, and funding structures for future global health experiences	
**Section**	**Content**
Reverse culture shock	What is culture shock?
	Preparing to return home
	The honeymoon
	Readjustment and adaptation

Reflecting	Reflecting
	Reflection exercises

Relationships	Old and new friends

Health issues	Feeling physically ill upon your return
	Feeling mentally ill upon your return

Future work	Staying involved
	Your future career
	Mentoring others

Course summary	Take home points
	Staying connected

Each part ends with a course summary, posttest, and evaluation. Upon obtaining a passing score of 70% or higher on each posttest, participants may receive a verified certificate from edX (for a $50 fee) or a Credly [45] digital credentialing badge free of charge.

Narrated videos of learners’ personal experiences that relate to the topic are accompanied by text narratives, case studies, practice questions, or a faculty-moderated forum discussion. Sample material is shown in Boxes 1 and 2.

Course participants provided demographic information upon enrollment. An integrated pre- and posttest (consisting of multiple choice questions) assessed knowledge of learners before and after each part of the course. Course evaluations were solicited [46] and used for the 2017 course revision.

Demographics were reported using descriptive statistics. A visual inspection of the histograms and QQ plots for the difference in averages indicated a normal distribution of scores for all three parts of the course. Average pre- and posttest scores of the three parts were compared using a paired *t*-test.

The authors’ Institutional Review Board deemed this study exempt.

## Results

Demographics of course participants from 26 October 2015 to 31 December 2016 are shown in Table 4.

**Table 4.** Demographics of PGGH learner enrollment 26/10/15–31/12/16^+^.

	Part 1	Part 2	Part 3
Total enrollment	5935	2056	1244
Countries represented	163	130	105
% International^++^	68.40%	70.50%	71.4%
**Country (%)**			
United States/Canada	35.3	29.5	31.8
India	5	5.3	8.8
United Kingdom	4	3.7	3.8
Other	55.7	61.5	55.6
Median age (years)	30	30	31
% Female	54.90%	52.30%	48.00%

*^+^*26 October 2015–31 December 2016. *^++^*Not from USA.

US-based participants comprised 29–31% of learners, with significant participation by other countries. The overall course completion rate was 4–32%, Overall participation and the percentage of female participants decreased in Parts 2 and 3 (Table 4). Mean pre- and posttest score difference for the course components are shown in Table 5.

**Table 5.** Mean difference in PGGH pre- versus post-test scores (2016 and 2017).

Part	*n*	Possible points	Mean pre-course score (SD)	Mean post-course score (SD)	Mean test score difference (95% CI)	*p* Value
**2016**						
Part 1	109	29	60.6% (20.9)	82.3% (17.8)	21.7% (17.8–25.6)	<0.001
Part 2	56	33	57.7% (21.2)	67.8% (23.9)	10.1% (2.8–17.4)	0.008
Part 3	29	10	76.9% (17.3)	91.4% (18.3)	14.5% (6.6–22.3)	<0.001
**2017**						
Part 1	213	31	71.4% (20.7)	87.8% (15.4)	16.5% (14.9–18.7)	<0.001
Part 2	173	40	65.8% (11.6)	81.7% (10.4)	16.0% (14.3–17.6)	<0.001
Part 3	123	10	77.0% (17.2)	92.5% (12.1)	15.5% (12.9–18.1)	<0.001

Post-course feedback was collected on REDCap surveys [46]. Learners expressed interest in faculty-moderated discussion forums, in an interactive map to promote learner networking, and in additional material for trainees coming *from* low-resource settings. The course was rated at 4.5 stars, which is comparable to learner ratings of other edX courses [47].

## Discussion

Nearly 6000 learners from 163 different countries have utilized *PGGH*. While this number is greater than any previous reports, the typical MOOC may enroll up to 25,000 learners [48]. *PGGH* was intended for medical trainees interested in GH; this audience is comparatively smaller than the general audience of mainstream MOOCs. At this time, we are not able to assess how many of these learners participated in a rotation. However, we are able to follow our own institutions’ students and residents. To our knowledge, 10 academic institutions currently require the course of their learners before GH experiences.

There was a higher than expected enrollment of international learners. While we were unable to explore the motivations and experiences of this group of learners, previous studies indicate that learners from high-, middle-, and low-income countries have the same motivations, including opportunities to experience different health-care systems, resource-different settings, and cultural exposure [33]. However, the preparation needs of international learners rotating to high-income countries are not the same as those of US learners rotating to LMICs. International learners may have felt that the original course content, primarily focused on learners from high-income countries, was not appropriate for them. The enrollment of international learners illustrates the accessibility of MOOCs and creates an opportunity for bidirectional engagement among learners. Going forward, the inclusion of content specifically for international learners on GH experiences in high-income countries in all parts of the 2017 revised course will support their continued participation.

As may be expected, there was a decline in participation over the three parts of the course. Our course completion rate is comparable to reported median completion rates of participants in other open-access free-of-charge MOOCs (5–36% depending on the participants’ intentions) [49]. For our course and in comparison, participants paying for a ‘verified certificate’ had a higher % completion rate. From the data collected, we are not able to determine the characteristics of learners who did not complete all three parts of the course, but the proportion of international learners remained relatively unchanged. Before 2017, learners took each part as a separate edX course; this may have contributed to the decline in learners from Part 1 to Part 3. The 2017 update presents Parts 1–3 within the same edX course and learner attrition is improved, as shown in Table 5. Both US and international learners may have decided against or delayed their participation in a GH experience leading to a decline in the numbers of learners completing Parts 2 and 3.

This study had some limitations. At this time, due to limited demographic information on the edX platform, we do not have very specific data for the entire learner body of over 5000; however, we do know how many of our own institutions’ students and residents have gone on a rotation after taking the course. Also, the fact that the course evaluation and pre- and posttests are optional impacted completion rates; this makes it difficult to draw conclusions. Additionally, thus far, outcomes have been limited to self-perception and immediate retention of knowledge. While test scores improved in the ‘verified certificate’ subset of learners, we have not yet evaluated attitudinal or behavioral change directly or indirectly, the impact of the course on our learners’ GH experiences, or their interaction with host populations.

In addition, participation in the discussion forums by the all-volunteer faculty was limited following the initial release. To address this, a faculty moderation schedule was created to ensure continuous faculty moderation of the discussion forums. We anticipate that this will encourage learners to participate actively and stay engaged throughout the entirety of the course.

Next steps for the course include measuring long-term impacts on learners (e.g., on career choices) and on host populations (e.g., on sustainability of projects) by conducting surveys and interviews a few years post-course completion. In addition, we are establishing ‘cohorts’ that allow learners from one institution to interact with each other and their home institution mentors which will enable us to further evaluate short- and long-term outcomes.

## Conclusion

This is the first published descriptive evaluation of an open-access, online, free-of-charge MOOC focused on the preparation of learners participating in STEGH. *PGGH* provides open-access, standardized, interactive, timeline-based, preparation for learners wishing to complete a STEGH. In its first year, there was substantial interest in this course from US-based and international learners (5935 learners from 163 countries) and significantly improved posttest scores (*p* < 0.001) in the subset of 213 learners evaluated.

## Figures and Tables

**Box 1.** Sample self-reflection exercise from PGGH Part 1: purpose and motivation section.

The first step in pursuing a global health learning experience is to consider your motivations and goals. Why do you want to participate in a global health learning experience? **Please click ‘New Post’ and use the space below to list your top 3 motivations for pursuing a global health rotation** *Learners have the opportunity to reflect on this question* via *the discussion forum. After they have responded, they can view other learners’ responses and are led in guided self-reflection with the following discussion points* **After some reflection, did some of your motivations include?** *Desire to* serve others, teach, learn new skills, work towards worldwide social justice, give of yourself, reduce health disparities, save as many lives as possible (*idealism*), seek distraction and relief from unpleasant realities (*escapism*), assist with a specific need in an existing medical system, assist with a specific gap in an educational curriculum, improve clinical diagnostic skills *Belief* that such work is inherently worthwhile, that you are simply ‘cut out’ or have a ‘calling’ for work in global health, and that one must give of oneself to live a ‘good life’ (*eudemonia*)

**Box 2.** Sample case study from PGGH part 3: readjustment and adaptation section.

Melissa returns to school and is excited to see her classmates and friends again. However, she struggles with telling others about her experiences over the summer. They ask questions such as ‘How was Uganda?’ She struggles with a simple answer, but they do not seem interested in anything longer than a one word or sentence answer. She is struck by how much money her friends spend eating at restaurants. Thinking of how little money her NGO had to spend feeding so many hungry orphans, she feels guilty and selfish spending so much on herself As classes begin, Melissa feels bored by the daily routine of lectures and wonders why she is doing this and if it really matters compared to the work she was doing this past summer. She finds herself pulling away from friends and family, spending more time alone **Symptoms of reverse culture shock (*abbreviated***) Family, friends, and old routines bore you after your excitement and challenges abroad You’re irritated by inane questions about your experiences and answer sarcastically You feel that you can’t fully explain your experience or its importance You feel alienated and experience ‘reverse homesickness’ for your host country You constantly criticize your own culture in comparison with where you’ve been
